# Early life environment influences the trajectory of post-partum weight loss in adult female rats

**DOI:** 10.1016/j.rbmo.2018.12.002

**Published:** 2019-05

**Authors:** C.E. Aiken, J.L. Tarry-Adkins, T.J. Ashmore, S.E. Ozanne

**Affiliations:** a University of Cambridge Metabolic Research Laboratories and MRC Metabolic Diseases Unit, Institute of Metabolic Science, Addenbrooke's Hospital, Cambridge CB2 0QQ, United Kingdom; b Department of Obstetrics and Gynaecology, University of Cambridge, Box 223, The Rosie Hospital and NIHR Cambridge Comprehensive Biomedical Research Centre, Cambridge CB2 0SW, United Kingdom

**Keywords:** Adipose mass, Developmental programming, Inter-pregnancy weight gain, Metabolism, Post-partum weight, Rat

## Abstract

**Research question:**

The physiological processes of pregnancy and lactation require profound changes in maternal metabolism and energy balance. The timescale of metabolic reversion after pregnancy, in particular post-partum weight loss, is highly variable between individuals. Currently, mechanisms influencing post-partum metabolic recovery are not well understood. The hypothesis tested here is that, in common with other metabolic and obesity-related outcomes, capacity for post-partum weight loss is influenced by developmental programming.

**Design:**

Adult female Wistar rats exposed to a maternal low-protein diet *in utero* then weaned onto a control diet post-natally (recuperated group) were compared with controls. Adult females from both groups underwent pregnancy at 3 months of age. Weight changes and metabolic parameters during pregnancy and lactation were compared between control and recuperated groups, and also with non-pregnant littermates.

**Results:**

Pregnancy weight gain was not different between the control and recuperated groups, but post-partum recuperated animals remained significantly heavier than both post-partum control animals (*P*<0.05) and their non-pregnant recuperated littermates (*P*<0.05) at the end of lactation. Post-partum recuperated animals had more intra-abdominal fat mass (*P*<0.05) and higher serum triglyceride concentrations (*P*<0.01) than controls. Post-partum recuperated animals also had increased expression of *IL6, NRF2* and *ALOX12* (key regulators of inflammation and lipoxygenase activity) in the intra-abdominal adipose tissue compared with control groups.

**Conclusions:**

Mothers who themselves have been exposed to adverse early life environments are likely to have slower metabolic recovery from pregnancy than controls. Failure to return to pre-pregnancy weight after delivery predisposes to persisting sequential inter-pregnancy weight gain, which can represent a significant metabolic burden across a life course involving several pregnancies.

## Introduction

Healthy pregnancy requires major adaptations of maternal physiology to accommodate the needs of a growing fetus and to amass sufficient maternal energy reserves for delivery and lactation (Tan and Tan 2013). Key metabolic adaptations during pregnancy include increased adipose mass, reduced insulin sensitivity, increased propensity to fasting ketosis and changes in body fluid composition (Lowe and Karban, 2014, Lacroix et al., 2016, Staelens et al., 2016). A significant body of evidence suggests that an individual's response to the metabolic challenges of pregnancy can be viewed as a ‘stress test’ that reveals the individual's underlying propensity to metabolic dysfunction (Williams 2003). For example, if the physiological reduction in normal glucose tolerance (due to placenta-derived insulin-resistance promoting peptides) provokes frank gestational diabetes, then the chance of developing type 2 diabetes in later life is at least seven-fold higher than in women who were normo-glycaemic in pregnancy (Bellamy et al. 2009). Thus the metabolic response to pregnancy can be viewed as both a window onto future health and an important opportunity to intervene to improve health over the life course.

Over the course of a lifetime, failure to recover to baseline from the physiological challenges of each pregnancy can lead to gradual accumulation of fat mass and subsequent long-term metabolic derangement (Amorim et al., 2007, Lipsky et al., 2012). This cycle of inter-gestational weight gain leads to an increasingly higher body mass index (BMI) with each successive pregnancy, to the detriment of both the mother (Abrams et al. 2017) and potentially her subsequent offspring (Oostvogels et al. 2014). Evidence suggests that around half of women have regained their pre-pregnancy weight by 12 months post-partum (Sagedal et al. 2017) although this is highly dependent on factors such as maternal age, socio-economic status, pre-pregnancy BMI, stress and breast-feeding (Jarlenski et al., 2014, Endres et al., 2015, Straub et al., 2016). Trials in human pregnancy show that diet and exercise interventions can be effective in limiting gestational weight gain (Muktabhant et al. 2015) and that these interventions may also be partially effective in promoting post-partum weight loss and altering maternal dietary behaviour (Ehrlich et al., 2014, Horan et al., 2014, Patel et al., 2017, Sagedal et al., 2017). However, very little is known about the molecular mechanisms that influence the propensity to post-partum weight loss.

Risk of obesity in adult life is increased by exposure to a suboptimal intrauterine environment (Bouret et al. 2015). It has previously been demonstrated using a rat model that adult offspring exposed to a low-protein maternal diet during pregnancy followed by post-natal catch-up growth (a ‘recuperated’ group) are prone to later-life obesity (Cripps et al., 2009, Berends et al., 2013) and metabolic derangement (Martin-Gronert et al. 2008). The hypothesis tested here, therefore, is that an adult female that was exposed to a suboptimal early life environment (with resulting programmed energy and glucose handling deficits) might have a reduced capacity for metabolic recovery post-partum, in addition to an increased risk of obesity in later adulthood, compared with control counterparts.

The aetiology of post-partum adipose mass retention might also be explored by examining adipocyte gene expression in the immediate post-weaning period. Gene expression of key metabolic, endocrine and reproductive pathways may provide insight into the molecular pathways that regulate post-partum weight loss. In addition to quantifying intra-abdominal fat mass in the post-partum period, the aim was also to assay the relative expression levels of a panel of key candidate genes, including those involved in the inflammatory adipose response (*Il1, Il6, Tnfa, Il10*), oxidative stress response (*Hmox1, Xo, NfkB, Gp91^phox^*), lipoxygenase activity (*Alox12, Alox15*), macrophage infiltration (*Mcp1, Cd68*) and master transcriptional regulators thought to be involved in the pathogenesis of obesity (*Tgfb, Tnfa, Nrf2*). These genes were chosen based on (i) previous work on the effects of developmental programming on ovarian and adipose gene expression (Aiken et al. 2016); (ii) knowledge of programming mechanisms in other organ systems in the same recuperated animal model (Aiken et al., 2016, Tarry-Adkins et al., 2016); and (iii) relevant literature review based on searching the PubMed and Medline databases using the MeSH terms ‘Postpartum Period’ AND ‘Body Weight Change’.

It has previously been shown that there is a significant relationship between developmentally programmed obesity and reduced primordial follicular reserve (Aiken et al. 2016) in adult females. It is not currently known whether this is a result of reduced follicular endowment in the perinatal period or an accelerated decline in follicular reserve during reproductive life. Understanding whether primordial follicular reserve is further reduced after the metabolic and endocrine challenge of pregnancy and lactation could give important insight into the dynamics of reduced ovarian reserve and hence future reproductive potential in developmentally programmed animals.

The aim of this study was therefore to determine whether post-partum weight loss and metabolic recovery from pregnancy were significantly different between control animals and those with programmed energy and glucose handling deficits resulting from exposure to a suboptimal intrauterine environment.

## Materials and methods

### Experimental design

All animal experiments were approved by the University of Cambridge Animal Welfare and Ethical Review Board. All animal experiments were conducted in accordance with the British Animals (Scientific Procedures) Act (1986) and were compliant with EU Directive 2010/63/EU. The aim of the study was to test whether post-partum metabolic recovery and weight loss were impaired in adult females that had been exposed to a maternal low-protein diet *in utero*. Wistar rat dams (F0 generation, *n*=16) were fed a standard laboratory chow diet (20% protein) and fed *ad libitum* until pregnancy was confirmed through the observation of vaginal plugs. Pregnant animals were then randomly assigned to a 20% protein diet (control) or an 8% isocaloric low-protein (LP) diet (*n*=8 in each group), as described previously (Martin-Gronert et al. 2008). Both diets were purchased from Arie Blok (Woerden, The Netherlands). Pups born to LP-diet-fed dams were cross-fostered to control-fed mothers at post-natal day 3, in order to create ‘recuperated’ offspring. The control group was not cross-fostered in this study, in keeping with the previous studies using this model (Aiken et al., 2013, Aiken et al., 2015, Tarry-Adkins et al., 2018). Extensive experiments have previously been performed to ensure that the cross-fostering process does not alter the phenotype in the recuperated group (organ weights, glucose tolerance and insulin tolerance; data not shown). Each recuperated litter was culled to four female pups (F1 generation, recuperated group, *n*=8 litters) at random to maximize their plane of nutrition. This culling protocol exacerbates the effect of the prenatal nutritional intervention and maximizes catch-up growth. Thus, the model combines two paradigms: both nutritionally induced in-utero growth restriction and also accelerated post-natal growth, which are phenomena often observed together in low-birthweight human babies. The control group was the offspring of mothers fed the 20% protein diet during gestation and suckled by 20%-protein-fed dams during lactation (F1 generation, control group, *n*=8 litters). Each control litter was culled to eight female pups. After weaning, all first-generation offspring were maintained on standard laboratory chow fed *ad libitum*. At 12 weeks of age, two first-generation females from each litter were randomly selected. One F1 female from each F0 litter (*n*=8 in each group) was paired with a stud male and the day of mating confirmed by the presence of a vaginal plug. The other female from each litter was not mated and was maintained in standard conditions as described previously.

During pregnancy, serial body weights were obtained every 4 days from both the pregnant female and her non-pregnant littermate. After giving birth, the second-generation (F2) litters were all culled to eight pups (both F2 control and F2 recuperated groups) on day 3 and were all suckled by their own mothers. At day 3, the first post-natal body weights were obtained, and thereafter every 7 days until weaning. There was no significant difference in birthweight or litter size in the F2 offspring between experimental groups. Detailed phenotyping of the F2 generation of this cohort is described elsewhere (Aiken et al., 2015, Tarry-Adkins et al., 2018). At weaning of the second-generation litter (post-natal day 21), all first-generation females (*n*=8 post-partum mothers and their 8 non-pregnant littermates, in each of the control and recuperated groups) were fasted overnight and fasting blood glucose concentrations were determined using a glucometer (Hemocue; Angelholm, Sweden). The first-generation females were culled by carbon dioxide asphyxiation and cervical dislocation. At post-mortem, serum samples, ovaries, ovarian fat pads and other solid intra-abdominal organs were harvested and weighed fresh, immediately after dissection. One ovary from each animal was snap-frozen in liquid nitrogen and the other fixed in formalin/paraldehyde. The fixed ovaries were sectioned and subjected to haematoxylin and eosin (H&E) staining to ensure equal distribution of oestrous stages in each experimental group (data not shown). Sample analysis was performed using project codes to blind the investigators to the experimental groups. Seven samples per group were analysed at each time point, each sample representing a different litter. The sample size was determined via a power calculation based on the effect sizes seen in previous studies (Aiken et al., 2013, Aiken et al., 2015), using an alpha level of 0.05 to give a power of 0.8.

### Serum analyte measurements

Blood was obtained from the tail vein, collected in EDTA tubes and centrifuged for 3 min at 955 *g* at 4°C to isolate serum. Fasted blood glucose measurements were obtained using a glucose analyser (Hemocue, Angelholm, Sweden). The serum lipid profiles were determined using an auto-analyser (MRC MDU Mouse Biochemistry Laboratory, Addenbrooke's Hospital, Cambridge, UK). Serum leptin was measured using an enzyme-linked immunosorbent assay kit from Crystal Chem (Zaandam, The Netherlands), which was used according to the manufacturer's instructions.

### Primordial follicle counts

Fixed ovaries were processed for microscopy and the entire ovary sectioned at 8 μm. Every ninth section was stained with H&E for morphometric analysis (72 μm between analysed sections). Only follicles with a visible oocyte nucleus were counted, in order to avoid repeat counts of the same follicle (Bernal et al. 2010). Primordial follicles were identified morphologically by the presence of a single layer of flattened granulosa cells surrounding the oocyte (Picut et al. 2014). Total volume of each ovary was calculated (section areas × section thickness × number of sections) and the follicle count expressed as follicles/mm^3^ of ovarian tissue.

### Gene expression analysis

A panel of 15 candidate genes was developed to test which molecular pathways might be involved in post-partum metabolic recovery. RNA was extracted from snap-frozen para-ovarian fat pads using a miRNeasy mini kit (Qiagen, Hilden, Germany) following the manufacturer's instructions, with the addition of a DNaseI digestion step to ensure no genomic DNA contamination. RNA quantification was performed using a NanoDrop spectrophotometer (Nanodrop Technologies, Wilmington, DE, USA). RNA (1 μg) was used to synthesize cDNA using oligo-dT primers and M-MLV reverse transcriptase (Promega, Madison, Wisconsin, USA). Gene expression was determined using custom-designed primers (Sigma, Poole, Dorset, UK) and SYBR Green reagents (Applied Biosystems, Warrington, UK), as previously described (Tarry-Adkins et al. 2009). Quantification of gene expression was performed using a Step One Plus RT-PCR machine (Applied Biosystems, Warrington, UK). Equal efficiency of the reverse transcription of RNA from all groups was confirmed through quantification of expression of the housekeeping gene *ppia*, the expression of which did not differ between groups.

### Statistical analysis

All data were analysed using hierarchical linear models with a random effect for litter of origin. Maternal diet and pregnancy status were included as fixed effects. This structure accounted for the fact that post-partum and non-pregnant littermates are derived from a single pregnancy, and these data are therefore effectively paired and cannot be treated as fully independent. Multiple hypothesis correction testing was performed using the *P-*values obtained from the regression models, correcting for the false discovery rate (FDR). All body weights were expressed as a ratio of current weight/weight on study day 0. Data are represented as means ± SE. A value of *P* < 0.05 was considered statistically significant. All data analysis was conducted using the R statistical software package, version 2.14.1 (R Foundation for Statistical Computing, Vienna, Austria). In all cases, *n* refers to the number of litters.

## Results

### Peri-partum body weights

**Figure 1:**
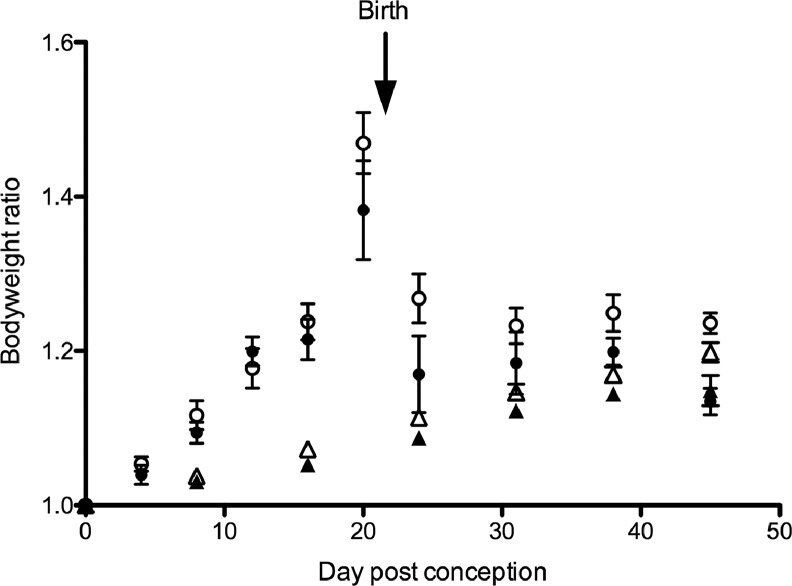
Body weights of adult females during pregnancy and lactation. All body weights are normalized to the weight at the start of the study. There was no significant difference in starting body weights between groups. Open circles, pregnant recuperated group; closed circles, pregnant control group; open triangles, non-pregnant recuperated group; closed triangles, non-pregnant control group. At the final study time point, the post-partum recuperated group was significantly heavier than any other study group.

There was no significant difference in average weight gain during pregnancy between the control and the recuperated group (Figure 1). At full term (day 20 after conception), the control and recuperated pregnant mothers were both approximately 40–50% heavier than their pre-pregnancy weights (control pregnant 1.38-fold ±0.06 versus recuperated pregnant 1.47-fold ±0.04). There was no difference in litter size or pup weight in the control versus the recuperated groups (full characterization of the F2 generation has been previously reported (Aiken et al. 2015)). However, by the end of lactation (day 45 post-conception and day 24 post-delivery), the recuperated post-partum mothers remained heavier than their control post-partum counterparts (control post-partum 1.13-fold ± 0.03 versus recuperated post-partum 1.24±0.01, p<0.01). Compared with their control non-pregnant littermates, the control post-partum mothers were not significantly heavier (control post-partum 1.13-fold ± 0.02 versus control non-pregnant 1.15-fold ± 0.02); however, the recuperated mothers were significantly heavier than their recuperated non-pregnant littermates (recuperated post-partum 1.24 ± 0.01 versus recuperated non-pregnant 1.19 ± 0.01, *P*<0.05).

### Organ weights

**Figure 2:**
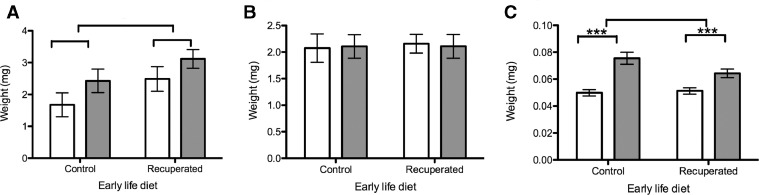
Organ weights in the post-partum period. Open bars, non-pregnant littermates; grey bars, post-partum females. (A) Para-ovarian fat pad, (B) uterus, (C) ovary . *P<0.05, ***P<0.001.

Para-ovarian fat pad weights were significantly higher in recuperated groups compared with controls (*P*<0.05) and non-significantly higher in the post-partum groups compared with the non-pregnant littermates (*P*=0.07) (Figure 2A). Overall, there was a significant interactive effect of recuperated status and post-partum status (*P*<0.05), indicating that intra-abdominal fat mass is more likely to be retained post-partum in recuperated animals. Uterine weights were not significantly different between any of the experimental groups (Figure 2B). This suggests that complete involution of the uterus had occurred by the time of study sampling, and thus the effects observed during the study were not the rapid dynamic changes of the early post-partum period, but a stable phenotype in the post-delivery phase.

### Serum analytes

There was no difference in blood glucose or serum insulin concentrations between any of the experimental groups (Table 1). There was a trend towards lower leptin in the post-partum groups (*P*=0.08), which likely represents lactational hypoleptinaemia, but no significant effect of recuperated versus control status (*P*=0.11). The relative suppression of leptin during lactation is a previously characterized effect, which drives a chronic hyperphagia to meet the metabolic demands of milk production (Smith et al. 2010).

**Table 1:** Concentrations of glucose, insulin and leptin in maternal serum by pregnancy and dietary group

	Control post-partum group	Control non-pregnant group	Recuperated post-partum group	Recuperated non-pregnant group	Effect of pregnancy	Effect of maternal diet
Blood glucose (mmol/l)	5.5 ± 0.4	5.3 ± 0.4	5.6 ± 0.3	5.4 ± 0.3	*P*=0.88	*P*=0.72
Serum insulin (ng/ml)	0.8 ± 0.04	0.8 ± 0.03	0.9 ± 0.06	0.8 ± 0.08	*P*=0.22	*P*=0.64
Serum leptin (ng/ml)	5.2 ± 0.71	7.1 ± 1.5	7.3 ± 0.1	10.5 ± 1.29	*P*=0.11	*P*=0.08

Values are mean ± SD, unless otherwise stated.

**Figure 3:**
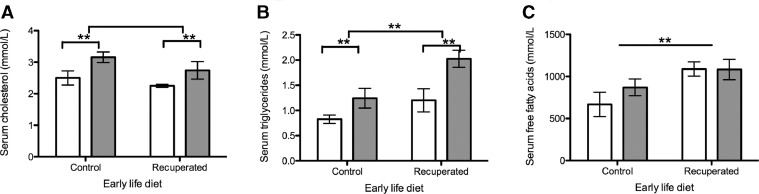
Maternal serum lipid profiles. All testing was performed following a period of overnight fasting. Open bars, non-pregnant littermates; grey bars, post-partum females. (A) Serum cholesterol (mmol/l), (B) serum triglycerides (mmol/l), (C) serum free fatty acids (mmol/l). **P<0.01.

There was a significant rise in serum cholesterol in both post-partum groups compared with their non-pregnant littermates (*P*<0.01), but no effect of recuperated status (Figure 3A). Fasting serum triglycerides were elevated in both the post-partum groups (*P*<0.01) and in the recuperated maternal dietary groups (*P*<0.01) (Figure 3B). Fasting free fatty acids were higher in the recuperated groups than in the controls (*P*<0.01) but unaffected by pregnancy status.

### Primordial follicle counts

**Figure 4:**
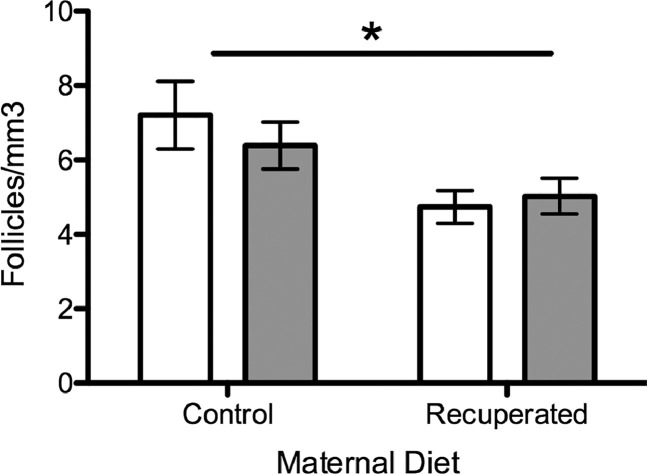
Primordial follicular reserve. Open bars, non-pregnant littermates; grey bars, post-partum females. Primordial follicular reserve was higher in the control than in the recuperated groups, but there was no significant difference by post-partum status. *P<0.05.

Ovary weights were significantly higher in the post-partum groups than in the non-pregnant littermates (*P*<0.001) but there was no difference between recuperated and control groups (Figure 2C). Primordial follicle counts per cubic millimetre of ovarian tissue were significantly higher in the control than in the recuperated groups (*P*<0.05), an effect that has previously been described in this model at 6 months of age (Aiken et al. 2013). There was no effect of having recently been pregnant on primordial follicle counts (Figure 4), which is to be expected, given the relatively short duration of the gestation period.

### Screening for differences in para-ovarian fat pad gene expression

A candidate screen on 15 genes was developed and the screening results were corrected for multiple hypothesis testing. There were no significant interactive effects between post-partum status and recuperated versus control status on expression levels of any of the candidate genes in para-ovarian adipose tissue (Table 2).

**Table 2:** Effect of pregnancy and maternal diet on gene expression in the para-ovarian fat pad of adult female rats

Gene	Pregnancy effect	Maternal diet effect	Interaction effect
*Ppia*	0.22	0.23	0.60
*Nrf2*	0.01*	0.16	0.59
*Hmox1*	0.14	0.46	0.28
*Xo*	0.56	0.35	0.62
*Alox12*	0.51	0.32	0.05
*NfkB*	0.97	0.09	0.13
*Il6*	0.02*	0.58	0.65
*I1b*	0.46	0.24	0.36
*Tnfa*	0.57	0.29	0.83
*Tgfb*	0.78	0.40	0.50
*Il10*	0.22	0.23	0.60
*Alox15*	0.41	0.62	0.29
*Mcp1*	0.06	0.05	0.83
*Cd68*	0.77	0.03*	0.27
*Gp91^phox^*	0.87	0.03*	0.51

All reported *P* values have been adjusted to take account of multiple hypothesis testing. *P<0.05.

#### Effect of recuperated versus control status on gene expression in para-ovarian adipose tissue

Recuperated versus control status in post-partum rats had a significant effect on expression levels of two candidate genes (Table 2). *Nrf2*, an important DNA-binding transcription factor that regulates mitochondrial biogenesis, was increased in recuperated adult females compared with controls (*P*<0.05). This is particularly interesting in light of other evidence that suggests *Nrf2* may be up-regulated in other maternal dietary models of developmental programming (Aiken et al. 2016). *Il6*, which is a major pro-inflammatory cytokine, was also up-regulated in adipose tissue in the recuperated group (*P*<0.05). This is important as it suggests not only that the total mass of intra-abdominal tissue is increased but also that one of its major detrimental effects, i.e. producing a phenotype of chronic inflammatory response, may be exacerbated in this developmental programming model.

#### Effects of recent pregnancy on gene expression in para-ovarian adipose tissue

Expression of *Cd68*, a marker of monocyte lineage, was elevated in animals that had recently been pregnant (*P*<0.05) (Table 2) compared with their non-pregnant littermates. Increased monocyte infiltration following pregnancy suggests increased inflammation in the adipose tissue. Furthermore, there was a significant increase in *Gp91^phox^* expression in the post-partum group (*P*<0.05), which potentially reflects an increase in oxidative stress in the para-ovarian adipose tissue.

## Discussion

This study shows that post-partum weight loss is influenced by early life exposure to an undernourishing intrauterine environment. It has previously been established in a variety of animal developmental programming models (Samuelsson et al., 2008, Samuelsson et al., 2013, Sun et al., 2014) and suggested by human epidemiological data (Finer et al., 2016, Mitanchez and Chavatte-Palmer, 2018) that adult females who have been exposed to adverse early life environments, e.g. a suboptimal maternal diet, are more likely to become obese in later life. This study advances the understanding of how pregnancy and post-partum recovery can be an important factor in influencing the propensity to obesity in developmentally programmed females.

In the rodent model, suboptimal in-utero nutrition significantly increased post-partum weight retention at the end of lactation. In particular, adult females exposed to a maternal low-protein diet *in utero* were on average 24% heavier than their own pre-pregnancy weights at the end of lactation (compared with 13% heavier in the control group). The study demonstrated that developmentally programmed animals that undergo pregnancy have a prolonged exposure to increased intra-abdominal fat mass compared with controls, a conclusion that is reflected both in direct measurement of the intra-abdominal fat pad and in elevated concentrations of serum triglyceride compared with control post-partum animals. Increased visceral adiposity is known to be detrimental to long-term health via a variety of mechanisms, including a chronic inflammatory response (Schlecht et al. 2016), which is in keeping with the observations here of increased *Cd68* and *Il6* gene expression. However, there was no evidence of any interaction between post-partum status and maternal diet in any particular gene expression pathway that could provide a clear insight into the molecular mechanism of post-partum weight retention in the programmed animals. However, the observed elevation in free fatty acids in the recuperated group is in keeping with adipose tissue insulin resistance.

Over 80% of women in the UK experience a viable pregnancy during their lifetime (ONS 2016). Despite the fact that pregnancy is an extremely common life event, the majority of female animals in developmental programming studies do not experience pregnancy as part of the longitudinal cohort structure. Even when animal models are bred to produce an F2 generation, it is rare that the post-partum outcomes of the F1 mothers are reported. However, it is increasingly understood that the physiological response to pregnancy and post-partum recovery can provide a useful window into later metabolic health (Drost et al., 2013, Visser et al., 2014). In common with gestational diabetes (Bellamy et al. 2009) and hypertension in pregnancy (Hermes et al. 2012), peri-partum changes in body weight could be a useful parameter to help identify individuals at high risk of later weight gain and poor cardiovascular and metabolic health. In addition to enabling identification of individuals at risk of obesity, post-partum weight retention may be an important factor in the causal mechanism of obesity in adult females (Ketterl et al. 2018). Post-partum weight loss and its inverse effect, inter-gestational weight gain, are becoming increasingly recognized as major determinants of women's health across the life course (Oteng-Ntim et al. 2018). A recent study found that only 20.7% of normal-weight women who received standard pregnancy care had regained their pre-pregnancy weight at 6 months post-partum, and that the average weight retention was 3.3 ± 3.5 kg (Phelan et al. 2011). Among those who had not returned to pre-pregnancy weight by 12 months, the average weight retention remained relatively stable, at 3 ±5.7 kg (Phelan et al. 2014). Over the course of several full-term pregnancies, each additional weight gain can thus accumulate to produce a significant metabolic burden (Hutcheon et al. 2017) in the mother, with additional potential detrimental consequences for children born from subsequent pregnancies. It is thus important to understand as much as possible about factors that influence post-partum weight loss. It is not possible to say whether the findings from this rodent population will be directly applicable to human pregnancy cohorts, hence the need for further study of factors influencing post-partum weight loss in human pregnancy cohorts.

A major advantage of the study design was the ability to compare each animal with a non-pregnant littermate, which allowed us to control for observable and non-observable factors related to the early life environment, and hence isolate specifically the implications of pregnancy. Furthermore, the experimental design controlled for the fact that these animals, early in their reproductive lives, were still themselves growing during the study period.

Limitations of the current study include the inability to follow these animals through a subsequent pregnancy and into reproductive senescence to measure whether post-partum weight loss directly influenced the extent of later-life weight gain. Unfortunately, this was beyond the scope of the design of the current cohort. Other limitations include the inability to vary the length of the lactation period, which may have a significant influence on post-partum weight loss, and the inability to study post-partum weight loss in mothers that did not suckle their offspring (which is common in human populations). This was not possible because of the need to standardize conditions for the F2 generation, whose outcomes have been reported elsewhere (Aiken et al. 2015).

In conclusion, the study has shown that exposure to a suboptimal early life environment influences the rate of post-partum weight loss. Understanding the factors that make post-partum weight loss more difficult represents the first key step towards developing interventions that can increase the percentage of women who have regained their pre-pregnancy weight by the start of the next pregnancy, and hence reduce their chances of obesity over their lifetime.

## References

[bib0001] Abrams B., Coyle J., Cohen A.K., Headen I., Hubbard A., Ritchie L., Rehkopf D.H. (2017). Excessive Gestational Weight Gain and Subsequent Maternal Obesity at Age 40: A Hypothetical Intervention. Am. J. Public Health.

[bib0002] Aiken C.E., Tarry-Adkins J.L., Ozanne S.E. (2013). Suboptimal nutrition in utero causes DNA damage and accelerated aging of the female reproductive tract. FASEB J..

[bib0003] Aiken C.E., Tarry-Adkins J.L., Ozanne S.E. (2015). Transgenerational Developmental Programming of Ovarian Reserve. Sci. Rep..

[bib0004] Aiken C.E., Tarry-Adkins J.L., Ozanne S.E. (2016). Transgenerational effects of maternal diet on metabolic and reproductive ageing. Mamm Genome.

[bib0006] Amorim A.R., Rossner S., Neovius M., Lourenco P.M., Linne Y. (2007). Does excess pregnancy weight gain constitute a major risk for increasing long-term BMI?. Obesity (Silver Spring).

[bib0007] Bellamy L., Casas J.P., Hingorani A.D., Williams D. (2009). Type 2 diabetes mellitus after gestational diabetes: a systematic review and meta-analysis. Lancet.

[bib0008] Berends L.M., Fernandez-Twinn D.S., Martin-Gronert M.S., Cripps R.L., Ozanne S.E. (2013). Catch-up growth following intra-uterine growth-restriction programmes an insulin-resistant phenotype in adipose tissue. Int. J. Obes. (Lond).

[bib0009] Bernal A.B., Vickers M.H., Hampton M.B., Poynton R.A., Sloboda D.M. (2010). Maternal undernutrition significantly impacts ovarian follicle number and increases ovarian oxidative stress in adult rat offspring. PLoS One.

[bib0010] Bouret S., Levin B.E., Ozanne S.E. (2015). Gene-environment interactions controlling energy and glucose homeostasis and the developmental origins of obesity. Physiol. Rev..

[bib0011] Cripps R.L., Martin-Gronert M.S., Archer Z.A., Hales C.N., Mercer J.G., Ozanne S.E. (2009). Programming of hypothalamic neuropeptide gene expression in rats by maternal dietary protein content during pregnancy and lactation. Clin. Sci. (Lond).

[bib0012] Drost J.T., van der Schouw Y.T., Maas A.H., Verschuren W.M. (2013). Longitudinal analysis of cardiovascular risk parameters in women with a history of hypertensive pregnancy disorders: the Doetinchem Cohort Study. BJOG.

[bib0013] Ehrlich S.F., Hedderson M.M., Quesenberry C.P., Feng J., Brown S.D., Crites Y., Ferrara A. (2014). Post-partum weight loss and glucose metabolism in women with gestational diabetes: the DEBI Study. Diabet. Med..

[bib0014] Endres L., K. Hub., McKinney C., Plunkett B., Minkovitz C.S., Schetter C.D., Ramey S., Wang C., Hobel C., Raju T., Shalowitz M.U., H. Community Child Health Network of the Eunice Kennedy Shriver National Institute of Child and D. Human (2015). Postpartum weight retention risk factors and relationship to obesity at 1 year. Obstet. Gynecol..

[bib0015] Finer S., Iqbal M.S., Lowe R., Ogunkolade B.W., Pervin S., Mathews C., Smart M., Alam D.S., Hitman G.A. (2016). Is famine exposure during developmental life in rural Bangladesh associated with a metabolic and epigenetic signature in young adulthood? A historical cohort study. BMJ Open.

[bib0016] Hermes W., Ket J.C., van Pampus M.G., Franx A., Veenendaal M.V., Kolster C., Tamsma J.T., Bloemenkamp K.W., Ponjee G., van der Hout E., Ten Horn H., Loix S., Mol B.W., de Groot C.J. (2012). Biochemical cardiovascular risk factors after hypertensive pregnancy disorders: a systematic review and meta-analysis. Obstet. Gynecol. Surv..

[bib0017] Horan M.K., McGowan C.A., Gibney E.R., Donnelly J.M., McAuliffe F.M. (2014). Maternal diet and weight at 3 months postpartum following a pregnancy intervention with a low glycaemic index diet: results from the ROLO randomised control trial. Nutrients.

[bib0018] Hutcheon J.A., Chapinal N., Bodnar L.M., Lee L. (2017). The INTERGROWTH-21st gestational weight gain standard and interpregnancy weight increase: A population-based study of successive pregnancies. Obesity (Silver Spring).

[bib0019] Jarlenski M.P., Bennett W.L., Bleich S.N., Barry C.L., Stuart E.A. (2014). Effects of breastfeeding on postpartum weight loss among U.S. women. Prev. Med..

[bib0020] Ketterl T.G., Dundas N.J., Roncaioli S.A., Littman A.J., Phipps A.I. (2018). Association of Pre-pregnancy BMI and Postpartum Weight Retention Before Second Pregnancy, Washington State, 2003-2013. Matern Child Health J.

[bib0021] Lacroix M., Battista M.C., Doyon M., Moreau J., Patenaude J., Guillemette L., Menard J., Ardilouze J.L., Perron P., Hivert M.F. (2016). Higher maternal leptin levels at second trimester are associated with subsequent greater gestational weight gain in late pregnancy. BMC Pregnancy Childbirth.

[bib0022] Lipsky L.M., Strawderman M.S., Olson C.M. (2012). Maternal weight change between 1 and 2 years postpartum: the importance of 1 year weight retention. Obesity (Silver Spring).

[bib0023] Lowe W.L., Karban J. (2014). Genetics, genomics and metabolomics: new insights into maternal metabolism during pregnancy. Diabet. Med..

[bib0024] Martin-Gronert M.S., Tarry-Adkins J.L., Cripps R.L., Chen J.H., Ozanne S.E. (2008). Maternal protein restriction leads to early life alterations in the expression of key molecules involved in the aging process in rat offspring. Am J Physiol. Regul. Integr. Comp. Physiol..

[bib0025] Mitanchez D., Chavatte-Palmer P. (2018). Review shows that maternal obesity induces serious adverse neonatal effects and is associated with childhood obesity in their offspring. Acta Paediatr..

[bib0026] Muktabhant B., Lawrie T.A., Lumbiganon P., Laopaiboon M. (2015). Diet or exercise, or both, for preventing excessive weight gain in pregnancy. Cochrane Database Syst. Rev..

[bib0027] ONS. 2016 Office of National Statistics Statistical Bulletin “Births in England and Wales: 2016 Live births, stillbirths, and the intensity of childbearing measured by the total fertility rate.” Release 19th July 2017. https://www.ons.gov.uk/peoplepopulationandcommunity/birthsdeathsandmarriages/livebirths/bulletins/birthsummarytablesenglandandwales/2016.

[bib0028] Oostvogels A.J., Stronks K., Roseboom T.J., van der Post J.A., van Eijsden M., Vrijkotte T.G. (2014). Maternal prepregnancy BMI, offspring's early postnatal growth, and metabolic profile at age 5-6 years: the ABCD Study. J. Clin. Endocrinol. Metab..

[bib0029] Oteng-Ntim E.D., Mononen S., Sawicki O., Seed P.T., Bick D., Poston L. (2018). Interpregnancy weight change and adverse pregnancy outcomes: a systematic review and meta-analysis. BMJ Open.

[bib0030] Patel N., Godfrey K.M., Pasupathy D., Levin J., Flynn A.C., Hayes L., Briley A.L., Bell R., Lawlor D.A., Oteng-Ntim E., Nelson S.M., Robson S.C., Sattar N., Singh C., Wardle J., White S.L., Seed P.T., Poston L. (2017). Infant adiposity following a randomised controlled trial of a behavioural intervention in obese pregnancy. Int. J. Obes. (Lond).

[bib0031] Phelan S., Phipps M.G., Abrams B., Darroch F., Grantham K., Schaffner A., Wing R.R. (2014). Does behavioral intervention in pregnancy reduce postpartum weight retention? Twelve-month outcomes of the Fit for Delivery randomized trial. Am. J. Clin. Nutr..

[bib0032] Phelan S., Phipps M.G., Abrams B., Darroch F., Schaffner A., Wing R.R. (2011). Randomized trial of a behavioral intervention to prevent excessive gestational weight gain: the Fit for Delivery Study. Am. J. Clin. Nutr..

[bib0033] Picut C.A., Remick A.K., Asakawa M.G., Simons M.L., Parker G.A. (2014). Histologic features of prepubertal and pubertal reproductive development in female Sprague-Dawley rats. Toxicol. Pathol..

[bib0034] Sagedal L.R., Sanda B., Overby N.C., Bere E., Torstveit M.K., Lohne-Seiler H., Hillesund E.R., Pripp A.H., Henriksen T., Vistad I. (2017). The effect of prenatal lifestyle intervention on weight retention 12 months postpartum: results of the Norwegian Fit for Delivery randomised controlled trial. BJOG.

[bib0035] Samuelsson A.M., Matthews P.A., Argenton M., Christie M.R., McConnell J.M., Jansen E.H., Piersma A.H., Ozanne S.E., Twinn D.F., Remacle C., Rowlerson A., Poston L., Taylor P.D. (2008). Diet-induced obesity in female mice leads to offspring hyperphagia, adiposity, hypertension, and insulin resistance: a novel murine model of developmental programming. Hypertension.

[bib0036] Samuelsson A.M., Matthews P.A., Jansen E., Taylor P.D., Poston L. (2013). Sucrose feeding in mouse pregnancy leads to hypertension, and sex-linked obesity and insulin resistance in female offspring. Front Physiol..

[bib0037] Schlecht I., Fischer B., Behrens G., Leitzmann M.F. (2016). Relations of Visceral and Abdominal Subcutaneous Adipose Tissue, Body Mass Index, and Waist Circumference to Serum Concentrations of Parameters of Chronic Inflammation. Obes. Facts.

[bib0038] Smith M.S., True C., Grove K.L. (2010). The neuroendocrine basis of lactation-induced suppression of GnRH: role of kisspeptin and leptin. Brain Res..

[bib0039] Staelens A.S., Vonck S., Molenberghs G., Malbrain M.L., Gyselaers W. (2016). Maternal body fluid composition in uncomplicated pregnancies and preeclampsia: a bioelectrical impedance analysis. Eur. J. Obstet. Gynecol. Reprod. Biol..

[bib0040] Straub H., Simon C., Plunkett B.A., Endres L., Adam E.K., McKinney C., Hobel C.J., Thorp J.M., Raju T., Shalowitz M. (2016). Evidence for a Complex Relationship Among Weight Retention, Cortisol and Breastfeeding in Postpartum Women. Matern Child Health J..

[bib0041] Sun B., Song L., Tamashiro K.L., Moran T.H., Yan J. (2014). Large litter rearing improves leptin sensitivity and hypothalamic appetite markers in offspring of rat dams fed high-fat diet during pregnancy and lactation. Endocrinology.

[bib0042] Tan E.K., Tan E.L. (2013). Alterations in physiology and anatomy during pregnancy. Best Pract Res. Clin. Obstet. Gynaecol..

[bib0043] Tarry-Adkins J.L., Aiken C.E., Ashmore T.J., Ozanne S.E. (2018). Insulin-signalling dysregulation and inflammation is programmed trans-generationally in a female rat model of poor maternal nutrition. Sci. Rep..

[bib0044] Tarry-Adkins J.L., Chen J.H., Smith N.S., Jones R.H., Cherif H., Ozanne S.E. (2009). Poor maternal nutrition followed by accelerated postnatal growth leads to telomere shortening and increased markers of cell senescence in rat islets. FASEB J..

[bib0045] Tarry-Adkins J.L., Fernandez-Twinn D.S., Chen J.H., Hargreaves I.P., Neergheen V., Aiken C.E., Ozanne S.E. (2016). Poor maternal nutrition and accelerated postnatal growth induces an accelerated aging phenotype and oxidative stress in skeletal muscle of male rats. Dis. Model Mech..

[bib0046] Visser S., Hermes W., Ket J.C., Otten R.H., van Pampus M.G., Bloemenkamp K.W., Franx A., Mol B.W., de Groot C.J. (2014). Systematic review and metaanalysis on nonclassic cardiovascular biomarkers after hypertensive pregnancy disorders. Am. J. Obstet. Gynecol..

[bib0047] Williams D. (2003). Pregnancy: a stress test for life. Curr. Opin. Obstet. Gynecol..

